# The relationship between the rate of melatonin excretion and sleep consolidation for locomotive engineers in natural sleep settings

**DOI:** 10.1186/1740-3391-4-8

**Published:** 2006-08-18

**Authors:** Gregory D Roach, Kathryn J Reid, Sally Ferguson, Drew Dawson

**Affiliations:** 1 Centre for Sleep Research, University of South Australia, City East Campus, Adelaide, SA 5000, Australia; 2 Department of Neurology, Northwestern University, Chicago, IL 60208, USA

## Abstract

**Background:**

The aim of the study was to examine the role that melatonin production plays in the regulation of sleep consolidation in a population of shiftworkers working and sleeping in their natural environments.

**Methods:**

253 locomotive engineers (249 male, 4 female, mean age = 39.7 years) participated in the study for a 2-week period whilst working their normal roster patterns. Participants recorded details for all sleep periods in a sleep diary and collected urine samples during each day's main sleep period. The samples were subsequently assayed for the metabolite of melatonin in urine, 6-sulphatoxymelatonin (aMT6s), and the rate of excretion during main sleep periods was calculated.

**Results:**

Separate one-way factorial ANOVAs revealed a significant effect of time of sleep onset on aMT6s excretion rate, sleep duration, and subjective sleep quality. Generally, the rate of aMT6s excretion was lower, sleep duration was shorter, and sleep quality was lower for sleeps initiated during the daytime than for sleeps initiated at night.

**Conclusion:**

Combined with previous studies linking melatonin production and sleep propensity, and others demonstrating the relationship between sleep consolidation and melatonin production in forced desynchrony protocols, the current results indicate that low production of melatonin may play a role in the poor consolidation of daytime sleep in natural sleep settings.

## Background

There are circadian rhythms of both sleep propensity (i.e. the ability to initiate sleep) and sleep consolidation (i.e. the ability to maintain sleep) that peak at around the time of the daily body temperature minimum (i.e. 03:00–05:00 h) and reach nadirs near the evening peak in the temperature rhythm [1-8]. There is also a circadian rhythm of melatonin production that is closely aligned with the daily body temperature cycle, such that it is low during the daytime, increases markedly in the evening, is high during the night-time, and drops at around dawn [9-11].

A number of studies indicate that the ability to initiate sleep is strongly associated with the daily rhythm of melatonin production. Importantly, the daily onset of melatonin production is related to the evening rise in sleep propensity, preceding it by approximately 100–120 minutes [12,13]. Furthermore, exogenous melatonin administered orally or by infusion increases subjective sleepiness and decreases latency to stage 1 and stage 2 sleep [14-18]. Taken together, these studies indicate that endogenous melatonin may have a direct effect on sleep regulation.

While a possible link between melatonin production and sleep propensity has been established, few studies have investigated the association between the circadian rhythms of melatonin production and sleep consolidation. Using a forced desynchrony protocol in one such study, Dijk *et al*. [19] found that sleep consolidation gradually deteriorated during the phase in the circadian cycle when melatonin secretion was low, and improved dramatically when sleep episodes coincided with the phase when melatonin secretion was high. This finding indicates that there may be a meaningful association between melatonin production and sleep consolidation, at least in controlled laboratory environments.

The aim of the current study was to examine the role that melatonin production plays in the regulation of sleep consolidation in a population of shiftworkers working and sleeping in their natural environments. It was hypothesised that the rate of melatonin excretion would be greater, sleep duration would be longer, and subjective sleep quality would be higher for sleep initiated during the night-time than for sleep initiated during the daytime.

## Methods

A total of 253 locomotive engineers (249 male, 4 female) gave written, informed consent to participate in the study as volunteers. Participants had a mean (±s.d.) age of 39.7 (±6.8) years and had been doing shiftwork for an average of 19.8 (±7.7) years. Participants did not receive any additional payment for participating in the study above their usual salary. The study was approved by the University of South Australia Human Research Ethics Committee.

Participants worked at one of fourteen rail depots in five Australian states. The depots chosen were representative of the varied work settings in the Australian rail industry and thus encompassed a wide range of working conditions and roster schedules. Participants drove electric or diesel locomotives; worked with another engineer or a conductor or drove alone; carried passengers, freight or coal; operated in rural or urban areas; and obtained rest at home or in barracks. In general, participants' rosters could be categorised as *irregular*. Work periods had a mean duration of 8.4 (±1.9) hours; 34.2% were shorter than 8 hours, 50.5% were 8–10 hours in duration, and 15.3% were longer than 10 hours. Furthermore, 43.9% of work periods began between 04:00–12:00 h, 34.0% began between 12:00–20:00 h, and 22.1% began between 20:00–04:00 h.

Data were collected at each rail depot in fourteen separate studies conducted successively over a 2-year period. In each study, data regarding participants' sleep patterns, and production of melatonin during sleep, were collected for fourteen consecutive days while the participants operated their normal roster pattern.

For every sleep period, participants recorded time of sleep onset, wake up time, duration of wake within sleep period, and subjective sleep quality in a sleep diary. Dependent measures derived from the sleep diaries were (i) sleep duration – the period between sleep onset time and wake up time, less awakenings, and (ii) sleep quality – participant's self-rating of sleep quality on a scale of 1 (very poor) to 5 (very good).

Production of melatonin during each day's main sleep period was inferred from urinary 6-sulphatoxymelatonin (aMT6s) excretion rates. All urine passed during a main sleep period was collected in a plastic bottle. At the end of the sleep period, participants voided urine into the bottle and marked the final level of urine on the bottle with a permanent marker. (The volume of urine produced during each sleep period was subsequently determined using these marks). Participants then transferred a 4 ml aliquot of urine by pipette to a 5 ml-sample tube. Samples were frozen as soon as practicable after collection. The concentration of aMT6s in the urine samples was subsequently determined by radioimmunoassay [20] using reagents obtained from Stockgrand Ltd. (Surrey, UK). Total excretion of aMT6s for each main sleep period was calculated by multiplying the assayed concentration by the volume of urine voided. The average rate of excretion was subsequently calculated by dividing the total amount of aMT6s excreted by the length of the sleep period.

Sleep quality, sleep duration, and the average hourly rate of aMT6s excretion during main sleep periods were each binned in 2-hour intervals depending on the time of sleep onset. The effects of time of sleep onset on each measure were determined using separate one-way factorial ANOVA.

## Results

**Figure 1:**
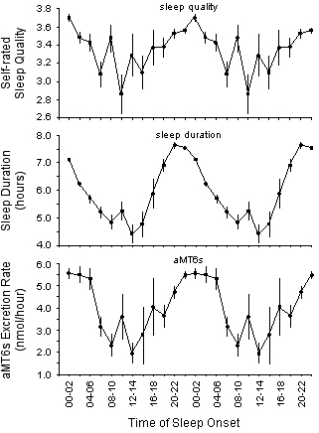
**Sleep quality, sleep duration, and aMT6s excretion rate. **Mean (±s.e.m.) subjective sleep quality (top panel), sleep duration (middle panel), and aMT6s excretion rate (bottom panel) as a function of time of sleep onset. Data are double-plotted on the x-axis.

Factorial ANOVA indicated that there was a significant effect of time of sleep onset on sleep quality (F_11,2694 _= 5.33, p < .0001). Specifically, there was a general decline in mean sleep quality from the daily peak for sleep initiated at 00:00–02:00 h to the daily minimum for sleep initiated at 10:00–12:00 h, and a general increase in sleep quality for sleep initiated between 10:00–12:00 h and 00:00–02:00 h (Figure 1, Table 1).

**Table 1:** Sleep quality, sleep duration, and aMT6s excretion rate (Mean ± s.e.m.) as a function of time of sleep onset

**Time-of-Day **	**Sleep Quality **	**Sleep Duration **	**aMT6s Excretion Rate **
**(2-hour bins)**	**(self-rated)**	**(hours)**	**(nmol/hour)**
0000–0200	3.70 (0.04)	7.13 (0.06)	5.57 (0.22)
0200–0400	3.48 (0.06)	6.22 (0.09)	5.51 (0.35)
0400–0600	3.43 (0.09)	5.69 (0.14)	5.33 (0.49)
0600–0800	3.08 (0.14)	5.19 (0.19)	3.15 (0.45)
0800–1000	3.47 (0.15)	4.84 (0.26)	2.31 (0.51)
1000–1200	2.86 (0.21)	5.24 (0.33)	3.60 (1.01)
1200–1400	3.29 (0.23)	4.42 (0.31)	1.95 (0.46)
1400–1600	3.10 (0.18)	4.77 (0.45)	2.77 (1.29)
1600–1800	3.37 (0.19)	5.87 (0.51)	4.06 (1.30)
1800–2000	3.38 (0.11)	6.91 (0.22)	3.64 (0.50)
2000–2200	3.53 (0.05)	7.64 (0.11)	4.71 (0.27)
2200–0000	3.56 (0.03)	7.54 (0.05)	5.48 (0.15)

Factorial ANOVA indicated that there was also a significant effect of sleep onset time on sleep duration (F_11,2959 _= 57.43, p < .0001). Specifically, there was a progressive fall in mean sleep duration from the daily peak for sleep initiated at 20:00–22:00 h to the daily minimum for sleep initiated at 12:00–14:00 h, and a sharp increase in sleep duration for sleep initiated between 12:00–14:00 h and 20:00–22:00 h (Figure 1, Table 1).

Finally, factorial ANOVA indicated that there was a significant effect of sleep onset time on the mean rate of melatonin production during sleep, as estimated by the rate of aMT6s excretion (F_11,2092 _= 4.99, p < .0001). Specifically, melatonin production was greatest for sleep initiated during the night-time (i.e. between 22:00–00:00 h and 04:00–06:00 h), and dropped to a daily minimum for sleep initiated at 12:00–14:00 h (Figure 1, Table 1).

## Discussion

Previous laboratory-based studies examining sleep consolidation have demonstrated that sleep duration and sleep quality are dependent on the time of sleep onset [21-23]. Specifically, sleeps are longest when initiated at around midnight, gradually become shorter as sleep onset is delayed beyond midnight into the early morning, and reach a minimum for sleeps initiated at around noon. Similarly, subjective sleep quality and sleep efficiency (i.e. the percentage of a sleep period spent sleeping) are highest when sleep begins in the late evening and lowest when sleep begins in the middle of the day.

In the current study, both sleep duration and subjective sleep quality varied depending on the time of sleep onset (Figure 1). Specifically, sleep duration was lowest for sleeps initiated at 12:00–14:00 h, and increased to a daily maximum for sleeps initiated at 20:00–22:00 h. Similarly, subjective sleep quality was lowest for sleeps initiated during the daytime, and peaked for sleeps initiated at 00:00–02:00 h. These results indicate that the sleep of locomotive engineers working and sleeping in their natural environments followed a similar pattern to that of participants in laboratory studies. In both cases, sleep initiated during the night-time tends to be longer and of better quality than sleep initiated during the daytime.

In addition to circadian rhythms of sleep duration and sleep quality, the current study also revealed a circadian rhythm of melatonin production. Specifically, the hourly rate of aMT6s excretion was greatest for sleeps that were initiated between 22:00–00:00 h and 04:00–06:00 h, and reached a minimum for sleeps that began at 12:00–14:00 h (Figure 1). Unfortunately, it is impossible to determine the extent to which the suppressive effects of light exposure prior to sleep may have contributed to the low rate of aMT6s excretion during the daytime in this sample. Nevertheless, the rhythm of melatonin production was closely associated, at least temporally, with the rhythms of both sleep duration and sleep quality. These results do not prove a causal relationship between melatonin production and sleep consolidation. However, when combined with previous studies linking melatonin production and sleep propensity [12-18], and evidence demonstrating the relationship between sleep consolidation and melatonin production in a forced desynchrony protocol [19], the current results do provide a further indication that melatonin may be involved in the regulation of sleep.

## Conclusion

Shiftworkers are in a constant struggle to obtain a sufficient amount of sleep between successive work periods, particularly between consecutive night shifts when they must attempt to sleep during the daytime [24]. The aim of the current study was to examine the role that melatonin production may play in this phenomenon in a population of shiftworkers who were working and sleeping in their natural environments. The results reveal that there were circadian rhythms of sleep quality, sleep duration, and aMT6s excretion that were temporally related, indicating that low production of melatonin may play a role in the poor consolidation of daytime sleep in natural sleep settings.

## Competing interests

The author(s) declare that they have no competing interests.

## Authors' contributions

GDR co-managed data collection, performed the statistical analyses, and was primarily responsible for drafting the manuscript.

KJR participated in the design of the study, co-managed data collection, and assisted in drafting the manuscript.

SF assisted in drafting the manuscript.

DD conceived of the study and participated in its design.

All authors read and approved of the final manuscript.
